# A242 EPIDEMIOLOGICAL ASPECTS, RISK FACTORS, AND CUMULATIVE PREVALENCE OF EXTRAINTESTINAL MANIFESTATIONS IN INFLAMMATORY BOWEL DISEASE: RESULTS FROM A 7,150 PATIENTS’ COHORT IN A TERTIARY CARE CENTER

**DOI:** 10.1093/jcag/gwad061.242

**Published:** 2024-02-14

**Authors:** E Lytvyak, A Montano Loza, B Halloran, F Hoentjen, A Mason, F Peerani, K Wong, R Fedorak, L Dieleman

**Affiliations:** University of Alberta Faculty of Medicine & Dentistry, Edmonton, AB, Canada; University of Alberta Faculty of Medicine & Dentistry, Edmonton, AB, Canada; University of Alberta Faculty of Medicine & Dentistry, Edmonton, AB, Canada; University of Alberta Faculty of Medicine & Dentistry, Edmonton, AB, Canada; University of Alberta Faculty of Medicine & Dentistry, Edmonton, AB, Canada; University of Alberta Faculty of Medicine & Dentistry, Edmonton, AB, Canada; University of Alberta Faculty of Medicine & Dentistry, Edmonton, AB, Canada; University of Alberta Faculty of Medicine & Dentistry, Edmonton, AB, Canada; University of Alberta Faculty of Medicine & Dentistry, Edmonton, AB, Canada

## Abstract

**Background:**

Canada is among the countries with a high burden of inflammatory bowel disease (IBD), including Crohn's disease (CD) and ulcerative colitis (UC). Extraintestinal manifestations (EIMs) incorporate a spectrum of systemic IBD-accompanying conditions that are associated with a poorer quality of life, higher disease activity, and increased need for IBD-related surgery and treatment escalation.

**Aims:**

We aimed to establish the all-time and cumulative prevalence of EIMs in a large cohort of IBD patients and assess the risk factors associated with EIMs in IBD.

**Methods:**

We conducted a retrospective cohort study of 7,150 IBD patients followed at the Division of Gastroenterology, University of Alberta, diagnosed between 1954–2022, with 159,026 person-years follow-up. Data were obtained via manual chart review, from electronic medical records and administrative reporting systems. The EIMs included ophthalmological, musculoskeletal, urogenital, hepatobiliary, dermatological, and pulmonary. Univariate and multivariate logistic regression models and cumulative prevalence curves with the Kaplan-Meier analysis were used.

**Results:**

Data of 3,910 CD and 3,240 UC patients (50.3% females, median age 48.0 (range 17-98 y.o.)) were analyzed. Over one-third of IBD patients (34.0%) had at least one EIM. The EIMs prevalence did not differ significantly between CD and UC (34.8% vs. 33.0%; p=0.112). In CD patients, the most common EIM was scleritis/episcleritis (10.9%), followed by nephrolithiasis (10.1%) and axial spondyloarthritis (7.3%). The UC patients most frequently had scleritis/episcleritis (10.3%), primary sclerosing cholangitis (8.4%) and nephrolithiasis (7.8%). In CD patients, age at diagnosis ≥40 y.o. (OR 1.77, 95%CI 1.33-2.37), disease duration ≥20 years (OR 2.28, 95%CI 1.62-3.21) and C-reactive protein≥8.0 mg/L (OR 1.46, 95%CI 1.01-2.12) were independent risk factors for EIMs (Fig.1a). Among UC patients, the following EIM risk factors were identified: disease duration ≥20 years (OR 1.63, 95%CI 1.19-2.22), obesity (OR 1.40, 95%CI 1.04-1.89), vitamin B_12_ deficiency (OR 1.64, 95%CI 1.12-2.40), and need for IBD surgery (OR 1.63, 95%CI 1.13-2.34) (Fig.1b). Cumulative probability of EIMs in CD vs. UC was 8% vs. 12%, at 10 years, 27% vs. 38% at 20 years, and 51% vs. 59% at 30 years since diagnosis (Log-rank, pampersand:003C0.001) (Fig.1c).

**Conclusions:**

Over one-third of IBD patients have at least one EIM and their pattern and cumulative prevalence varies substantially between CD and UC. It is important to be aware of the EIMs’ risk factors to recognize them early and provide adequate management aiming to decrease morbidity and mortality and improve the quality of life of IBD patients.

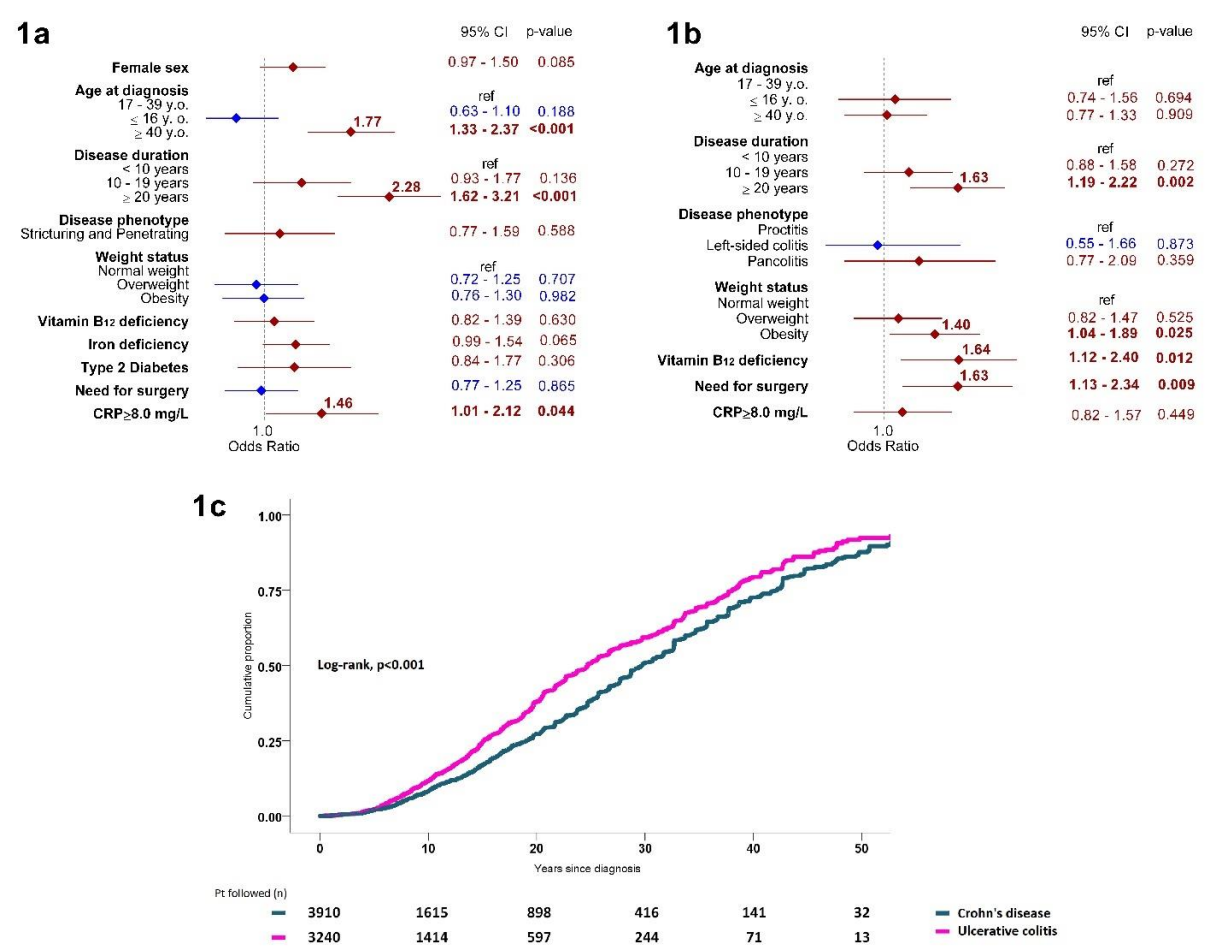

Fig. 1a. Associations between demographic, phenotypic and clinical IBD features and EIMs among CD patients - results of the multivariate logistic regression analysis.

Fig. 1b. Associations between demographic, phenotypic and clinical IBD features and EIMs among UC patients - results of the multivariate logistic regression analysis.

Fig 1c. Cumulative prevalence of EIMs in CD (grey line) vs. UC (pink line).

**Funding Agencies:**

None

